# The impact of structural diversity and parameterization on maps of the protein universe

**DOI:** 10.1186/1753-6561-7-S7-S1

**Published:** 2013-12-20

**Authors:** Daniel Asarnow, Rahul Singh

**Affiliations:** 1 Department of Computer Science, San Francisco State University, 1600 Holloway Ave, San Francisco, CA, 94132, USA; 2 Department of Computer Science, San Francisco State University, 1600 Holloway Ave, San Francisco, CA, 94132, USA; 3 Center for Discovery and Innovation in Parasitic Diseases, University of California, San Francisco, San Francisco, CA, USA

## Abstract

**Background:**

Low dimensional maps of protein structure space (MPSS) provide a powerful global representation of all proteins. In such mappings structural relationships are depicted through spatial adjacency of points, each of which represents a molecule. MPSS can help in understanding the local and global topological characteristics of the structure space, as well as elucidate structure-function relationships within and between sets of proteins. A number of meta- and method-dependent parameters are involved in creating MPSS. However, at the state-of-the-art, a systematic investigation of the influence of these parameters on MPSS construction has yet to be carried out. Further, while specific cases in which MPSS out-perform pairwise distances for prediction of functional annotations have been noted, no general explanation for this phenomenon has yet been advanced.

**Methods:**

We address the above questions within the technical context of creating MPSS by utilizing multidimensional scaling (MDS) for obtaining low-dimensional projections of structure alignment distances.

**Results and conclusion:**

MDS is demonstrated as an effective method for construction of MPSS where related structures are co-located, even when their functional and evolutionary proximity cannot be deduced from distributions of pairwise comparisons alone. In particular, we show that MPSS exceed pairwise distance distributions in predictive capability for those annotations of shared function or origin which are characterized by a high level of structural diversity. We also determine the impact of the choice of structure alignment and MDS algorithms on the accuracy of such predictions.

## Background

Understanding the molecular bases of protein structure and protein function is the preeminent challenge of molecular biology. One of the important problems in this context is that of obtaining a holistic description of protein architecture and to aid determination of structure-function relationships. In recent years, increasing availability of structural data has made it possible to rigorously investigate this question. However, even in the early days of molecular biology, when only a few thousand structures had been solved, researchers had espoused the importance of obtaining a grand view of the "universe" of all possible proteins [1,2]. To develop an intuition for this formulation, it is helpful to imagine every protein structure as a point in an abstract high dimensional fold space (hereafter called the protein structure space and abbreviated as PSS). Given a collection of proteins, such as the PDB, one can then ask a number of questions. For instance, do all the structures lie on a manifold in the PSS (i.e. is there a prominent shape to the distribution of structures)? How do functional characteristics map to specific regions of the PSS and vice-versa? How varied is the distribution of molecules in this space? The reader may note the fundamental nature of these questions and their relation to the dominant folding pathways, evolutionary processes, and physical constraints that interact to create all proteins.

A natural way to characterize a set of protein structures is through the set of pairwise distances computed for all proteins in the set using a suitable method for determining structure similarity. The set of all pairwise distances can then be used to map (define) a PSS by embedding the pairwise distances in a low dimensional space in a manner that is injective and minimally distorts the inter-structure dissimilarity information. In this paper we refer to this low dimensional representation as a map of the protein structure space (MPSS). In the context of creating such maps, the embedding methods that have been traditionally used include multidimensional scaling [1,3-5], correspondence analysis using reciprocal averaging [2], and principle component analysis [6]. Of these, multidimensional scaling has been the most widely used approach.

### Problem formulation

MPSS have been used to characterize an overall picture of protein space and predict annotations of protein structure. However, there has been little investigation of the effects of critical parameters on MPSS representations of protein space. Such parameters include the set of structures considered, choice of distance measure (i.e. the alignment and scoring methods), whether a degree of sparseness or a limit on maximum distance are present or imposed, and finally the choice of the method for computing the low dimensional embedding (projection). Furthermore, while a differential accuracy in prediction of functional annotation has been noted for particular annotations [4], no hypothesis has been put forward to explain this phenomenon. It is also unclear as to under what circumstances there is a consistent advantage in prediction of functional or other annotations using MPSS distances rather than distributions of pairwise structural differences.

In this paper we examine the influence of critical parameters on the construction of MPSS using multidimensional scaling (MDS), a widely employed method for low-dimensional projection. Our analysis includes the impact of specific measures of structural similarity and the influence of the specific algorithm for MDS (either "classical" or "iterative majorization," *vide infra*) on the MPSS. Further, we show that different groups of structures with common evolutionary origins are characterized by different levels of structural diversity, and that groups possessing high levels of structural diversity are not well captured by pairwise distances. In contrast, proximity within a MPSS predicts shared evolutionary origins of diverse groups with nearly the same efficacy as structurally self-similar groups. We also investigate the relative predictive capacities of MPSS generated using similarity scores obtained from the CE, Dali and FATCAT structure alignment algorithms. Finally, we investigate how this predictive capacity is altered by the choice of the multi-dimensional scaling algorithm.

### Data set

The set of protein structures used constitutes an essential parameter for MPSS construction because a MPSS localizes a structure in terms of its distance from the other structures. Given that we seek to characterize fold space globally in addition to multiple large structure families, we employ approximately 4,000 structures from the Nov. 2008 PDBSelect25, a list of non-redundant structures from the PDB, clustered at 25% sequence identity [7]. This set contains disparate structures representing the extents of the protein universe. Further, empirical studies indicate that subsampling, down to 1,000 or fewer structures, does not appear to qualitatively alter any of the results presented in this paper (data not shown).

## Methods

### Measurement of protein distances

The dissimilarity or distance between pairs of individual protein structures can be estimated using algorithms for pairwise alignment of protein structures. Such algorithms typically produce a similarity score as well as the alignment itself. For a set of *N *structures, a symmetric *N × N *matrix of similarity scores for all possible pairs can be constructed by performing the N(N+1)/2 unique, non-self pairwise alignments. Similarity scores must be inverted if they are to represent distances or dissimilarities. To avoid the introduction of bias by extremely high-scoring outliers, the similarity score *s_n _*with the *p*^th ^percentile rank is treated as a maximum similarity. The index *n *of the *p*^th ^percentile of *N *sorted similarity scores is computed using Equation (1). In turn, the enforced saturation of similarity scores implies the assumption of an "infinity distance" beyond which increasing distance does not indicate significant increase in real dissimilarity. Given pairwise similarity scores *s_i, j_*, for all pairs of structures *i *and *j*, the distances *δ_i, j _*are given by Equation (2).

(1) $$n=\frac{p}{100\times \left(N(N+1)/2+1\right)}$$

(2) $$\delta_{i,j}=\left\{\begin{array}{ccc} s_{n}-s_{i,j} & , & s_{n}>s_{i,j}\;,i\neq j\\ 0 & , & i=j\\ s_{n} & , & \;s_{n}\leq s_{i,j} \end{array}\right)$$

Different alignment methods employ distinct alignment algorithms as well as measures of overall alignment significance or pairwise structural similarity. To study the influence of alignment methods on the MPSS, we use three widely used methods: CE [8], Dali [9] and FATCAT [10]. Implementations of the three alignment methods are provided by the BioJava bioinformatics library [11] and by the DaliLite workbench [12]. Here we use the raw similarity scores determined by these algorithms rather than transformed scores representing statistical significance or other measures of saliency. Whether or not scores are transformed, and if so how (e.g. statistical standardization or protein length normalization), constitutes an additional parameter, consideration of which is beyond the scope of the present work.

### Low-dimensional projection using classical MDS

Let *d_ij_*(***X***) be the Euclidean distance between points *i *and *j *in a *d*-dimensional configuration ***X ***containing *N *points, then the matrix of inner products ***A = XX***^*T *^is derived following Equations (3) and (4). The scaling problem consists in solving Equation (4) for a configuration ***X***, with empirical distances ***δ ***substituted for the *d_ij_*(***X***).

(3) $$d_{ij}^{2}\left(X\right)=\overset{d}{\underset{k=1}{\sum }}X_{ik}^{2}+\overset{d}{\underset{k=1}{\sum }}X_{jk}^{2}-2\overset{d}{\underset{k=1}{\sum }}X_{ik}X_{jk}$$

(4) $$A_{ij}={\left(XX^{T}\right)}_{ij}=\overset{d}{\underset{k=1}{\sum }}X_{ik}X_{jk}=-\frac{1}{2}\left(d_{ij}^{2}\left(X\right)-\overset{d}{\underset{k=1}{\sum }}X_{ik}^{2}-\overset{d}{\underset{k=1}{\sum }}X_{jk}^{2}\right)$$

Note that the diagonal summation terms may be obtained directly from ***δ ***as the sum of row, column and grand means. Now, consider some configuration ***X***, the coordinates of which are normalized by their sums squared, yielding coefficients placed in a diagonal matrix ***λ****^1/2 ^*and orthonormal vectors ***v***. The inner product matrix ***A ***in terms of ***λ***^*1/2 *^and ***v ***is given by Equation (5) (recall that the inverse and the transpose of an orthogonal matrix are equivalent). However, this is equivalent to Equation (6), which we recognize as specifying the Eigen-structure of ***A***.

(5) $$A=XX^{T}=\left(\lambda^{1/2}v\right){\left(\lambda^{1/2}v\right)}^{T}=v\lambda v^{T}=v\lambda v^{-1}$$

(6) Av=λv

The *k*^th ^coordinate of the *i*^th ^point is found by Eigen-decomposing ***A ***and applying Equation (7):

(7) $$X_{ik}=\sqrt{\lambda_{k}v_{ik}}\;,\;\;k\in \left[1,d\right]$$

Since we are interested in dimensionality reduction we choose *d *≪ *N *even though in general ***A ***possesses *N-1 *non-zero eigenvalues. This procedure inevitably discards information; the proportion of information (i.e. displacement between various points) discarded is equivalent to the square root of the sum of the unused eigenvalues divided by the sum of all eigenvalues. Further information regarding classical MDS, including detailed proofs of the above, may be found in [13,14].

### Scaling by majorizing a complex function (SMACOF)

The sum-squared deviation, or stress, between a distance matrix ***δ ***and the distance distribution of a *d*-dimensional configuration of points ***X ***is defined by Equation (8):

(8) $$\sigma \left(X\right)=\underset{i<j\leq N}{\sum }w_{ij}{\left(d_{ij}\left(X\right)-\delta_{ij}\right)}^{2}$$

Where *w_ij _*is the weight given to the relationship between points *i *and *j*. Classical MDS does not, in general, arrive at the set of point with the least stress given the observed distances, because the relative separation or proximity of points in dimensions higher than *d *is not preserved. In general, it is possible for a minimization procedure targeting stress to "fold" these higher dimensional separations into the *d *dimensions of the point cloud.

The stress between a distance matrix and a point configuration can be minimized using iterative majorization, a procedure from convex analysis with strong convergence and speed guarantees [15]. This approach takes a configuration of points and perturbs it so as to reduce the stress induced by the original configuration. In brief, the new position ${\bar{x^{\prime }}}_{i}$ of a point *i *given its current position $\bar{x_{i}}$ and the current positions of all other points $\bar{x_{j}}$, is set to be the weighted average position of all the points, plus a weighted perturbation specified by the stress specifically induced between points *i *and *j*, as in Equation (9).

(9) $$\overset{⇀}{{x_{i}}^{\prime }}=\frac{1}{\Sigma_{j}^{N}w_{ij}}\overset{N}{\underset{j}{\sum }}w_{ij}\left(\overset{⇀}{x_{j}}+\frac{\delta_{ij}\left(\overset{⇀}{x_{i}}-\overset{⇀}{x_{j}}\right)}{∥\overset{⇀}{x_{i}}-\overset{⇀}{x_{j}}∥}\right)$$

The simple form of Equation (9) is due to a convex relaxation which ensures minimization of Equation (8) can be reduced to that of a simple quadratic (see [15] for proof). We use coordinates obtained from classical MDS as the initial configuration and iteratively evaluate Equation (9) until the relative change in stress falls below a set threshold. In this work, the threshold for relative change is taken to be 0.001, and all weights *w_ij _*are taken to be 1.

## Results and discussion

### Influence of alignment methods on the shape of MPSS

**Figure 1:**
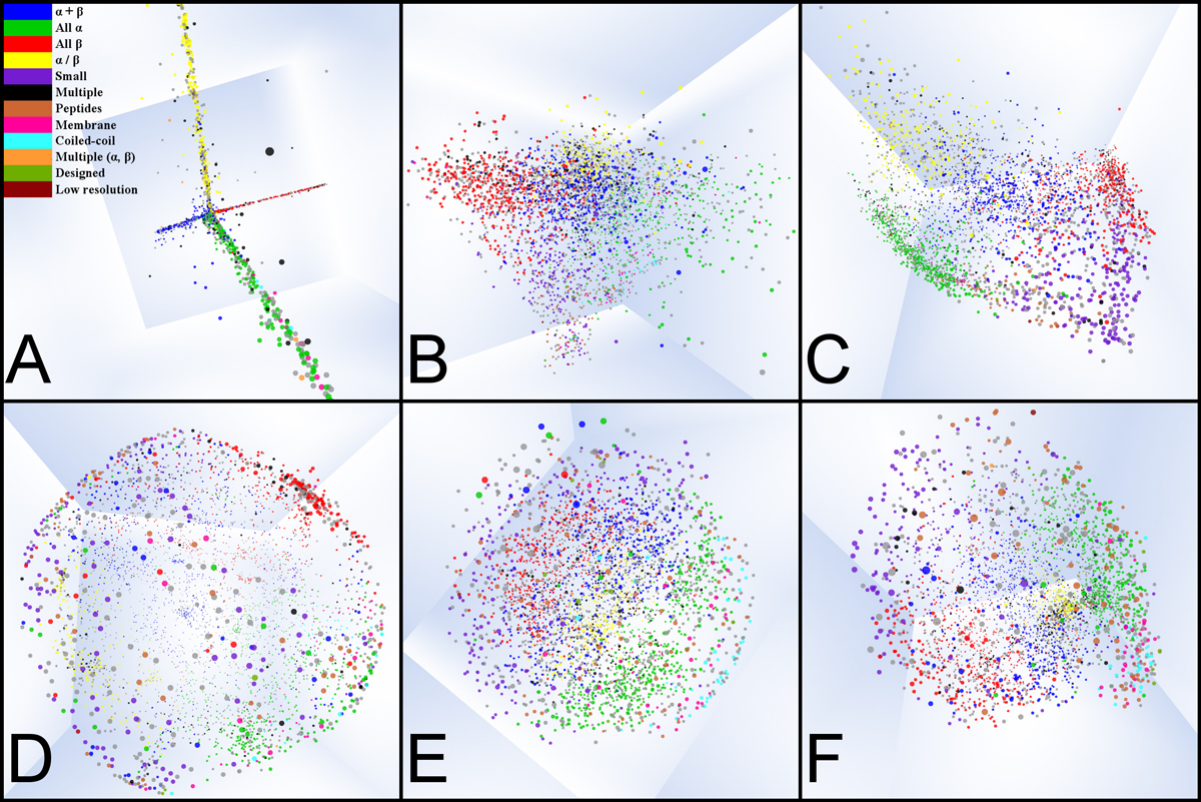
**MPSS by alignment algorithm and MDS method**. (A)-(C) MPSS using classical MDS for Dali, CE and FATCAT, respectively. (D)-(F) MPSS using SMACOF for Dali, CE and FATCAT, respectively. Each point represents a single structure, colored by SCOP class.

MPSS drawn using classical MDS and distances from Dali, CE and FATCAT are shown in Figure 1A-C. Each point represents a protein structure, colored by SCOP class. The particular shape of the Dali-based MPSS is caused by Dali's stringent filter for the significance of an alignment. For our data set, Dali returned an alignment score for just 0.05% of about 8 million pairs. This sparseness permits only the highest scoring alignments to influence the final configuration of the MPSS coordinates. MPSS produced with FATCAT and CE have a roughly pyramidal shape with small proteins and peptides located at the apex, and all α, all β, α+β and α/β classes forming the sides. MPSS drawn using SMACOF are shown in Figure 1D-F. While each SCOP class remains spatially segregated, the points are deformed towards a somewhat spherical arrangement. All MPSS were constructed using a distance cutoff percentile of 99.95%.

### Accuracy of annotation inference using alignment and MPSS distances

Pairwise alignment scores or distances within a MPSS can be used to predict annotations of common evolutionary origin in CATH and SCOP. A simple approach for this involves selecting a threshold distance and predicting any proteins separated by a distance equal to or less than the threshold to be related. In order to investigate the behavior of this distance based classifier, we construct Receiver Operating Characteristic (ROC) curves by computing the true positive rate (TPR or Sensitivity) and false positive rate (FPR or 1 - Specificity) of classification as the threshold distance is varied across a range. Such ROC plots permit comparison of the accuracy of alternate classifiers; a uniformly random classifier is indicated by a line at an angle of 45 degrees, while superior classifiers bend towards the upper left corner of the graph.

Application of the ROC approach to pairwise alignment or MPSS data is complicated by the variation of structural diversity between groups. If a single distance threshold is applied regardless of within-group distance distribution, a systematic bias will be introduced, resulting in increased false positives for structurally homogeneous groups and increased false negatives for diverse groups. We avoid the bias of a single threshold by directly varying percentile ranking, which is used to compute a unique threshold for each group. The same percentile rank will give a lower threshold for self-similar groups and a higher one for diverse groups. The threshold *T *for a group given the *p*^th ^percentile value *n *of all N sorted distances *d_i _*is determined by using Equation (1) and setting $T=d_{n}$. For this group and percentile rank, any structures found within the cutoff distance *T *of each other are considered positive matches. Small groups, possessing fewer than ten members, are excluded from consideration as small collections are likely to have low signal-to-noise ratios. The groups which meet this criterion within our data set are listed in Table 1.

**Table 1:** Selected annotation terms.

SCOP Superfamilies				SCOP Families				CATH Superfamilies			
**Name**	**No**.	**Name**	**No**.	**Name**	**No**.	**Name**	**No**.	**Name**	**No**.	**Name**	**No**.

"winged helix" DNA binding domain‡	49	nucleic acid binding proteins*	22	transmembrane helical fragments*	17	fibronectin type III**^‡^**	17	"winged helix" repressor DNA binding domain*	49	zinc/RING finger domain, C3HC4 (zinc finger)*	17

thioredoxin-like‡	38	EF-hand‡	20	canonical RBD	20	homeodomain*	10	immunoglobins	47	SH3 domains*	16

PH domain-like‡	29	transmembrane helical fragments*	17	pleckstrin homology domain (PH domain) ‡	15	globins**^‡^**	9	P-loop containing nucleoside triphosphate hydrolases**^‡^**	31	EF-hand**^‡^**	17

NTF2-like	26	scorpion toxin-like*	17	SH3 domain*	12	V set domains (antibody variable domain-like)	12	Glutaredoxin	31	homeodomain-like*	16

homeodomain-like	30	RNA binding domain (RBD) *	20	ubiquitin-related	11	thioltransferase	9	Rossmann fold**^‡^**	20	Phosphatidylinositol 3-kinase Catalytic Subunit	15

P-loop containing nucleoside triphosphate hydrolases**^‡^**	27	omega toxin-like*	16	N-actyl transferase (NAT)**^‡^**	11	retinol binding protein-like**^‡^**	9	nucleic acid binding proteins*	20	nuclear transport factor 2**^‡^**	14

ubiquitin-like	24	E set domains	17	spider toxins*	11	cold shock DNA binding domain-like*	13	aldolase class I**^‡^**	19	Pleckstrin-homology domain (PH domain)/Phosphotyrosine-binding domain (PTB)**^‡^**	14

immunoglobin	28	S-adenosyl-L-methionine-dependent methyltransferases	16	monodomain cytochrome c	10			lipocalin**^‡^**	17	globins**^‡^**	13

Diverse	92	All	379	Diverse	63	All	186	Diverse	118	All	356

Self-similar	143			Self-simlar	61			Self-similar	145		

Diverse and homogeneous groups are selected by inspecting within-group FATCAT distance distributions and taking those groups with the greatest and least mode distances, respectively. Only groups containing ten or more members were considered. Diverse groups are denoted by * and self-similar groups by **‡**.

**Figure 2:**
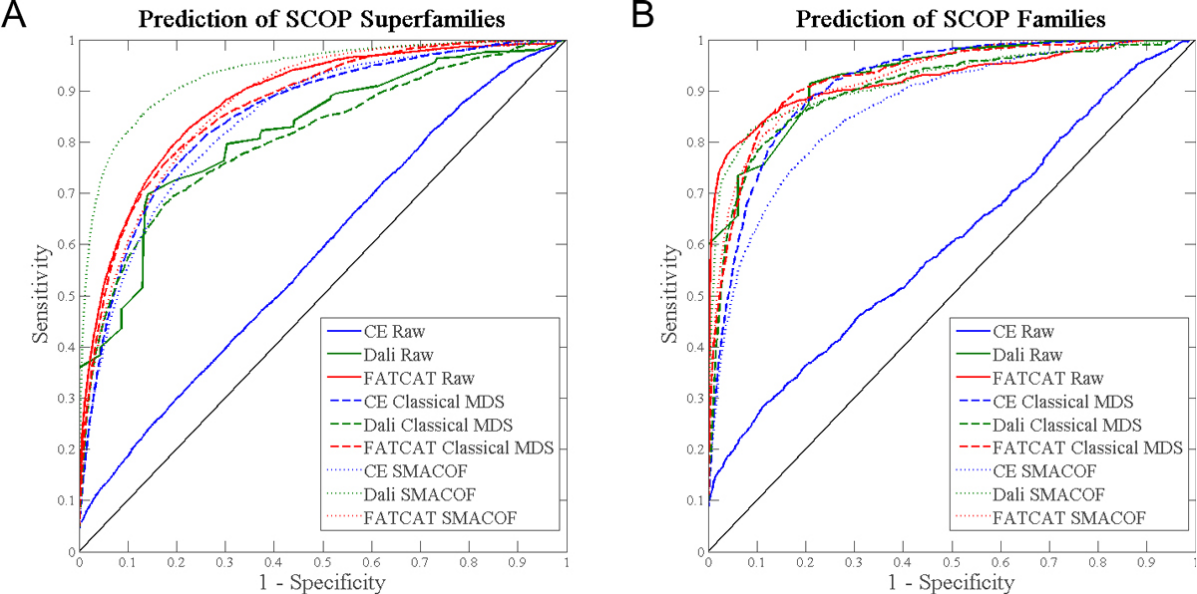
**Prediction of SCOP annotations by pairwise distances and MPSS proximity**. ROC curves indicate the performance of a classifier. The solid black diagonal represents a random classifier; better performing classifiers bend towards the upper left of the plot. Parts (A) and (B) contain ROC curves for prediction of membership in SCOP superfamiles and SCOP familes, respectively. Curves are given for each aligner (CE, Dali, FATCAT), using either raw distances, or proximity within MPSS constructed with either classical MDS or stress majorization. In particular, the plots demonstrate that MPSS distances are never significantly worse than pairwise alignment distances.

**Figure 3:**
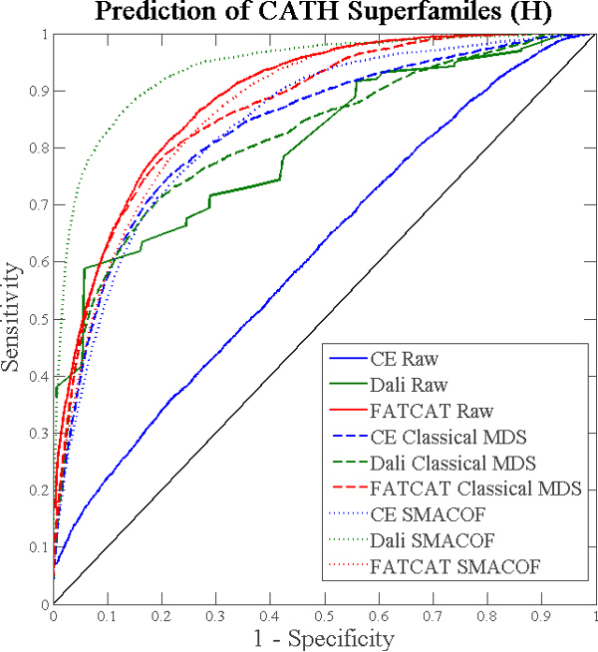
**Prediction of CATH annotations by pairwise distances and MPSS proximity**. As Figure 2, but for CATH homologous superfamilies ("H").

Figures 2 and 3 present ROC curves indicating the relative classification accuracy of pairwise alignment distances and proximity within MPSS constructed using classical scaling or stress majorization, for each annotation type. Importantly, the plots show that MPSS proximity is never significantly worse than pairwise alignment distances for predicting an annotation of common evolutionary origin. The plots also show that there are significant differences between structure alignment algorithms. FATCAT and Dali both take into account flexibility or relative displacement of similar components within structures, while CE produces a rigid local alignment only. As such, FATCAT and Dali are superior at detecting the distant or deformed structural similarities which might convey a functional relationship and/or shared origin. Furthermore, while raw CE distances do not perform well, low-dimensional projection with MDS rescues the classification ability of CE. Thus, it is shown that while transformation with MDS does not significantly hamper prediction of annotation, it in fact greatly improves prediction in some circumstances.

### Impact of SMACOF

The data of Figures 2 and 3 permits a direct comparison between MPSS produced using classical MDS and those which have been subject to minimization of the stress between pairwise alignment distances and MDS coordinates using SMACOF. In most cases, SMACOF appears to result in a small improvement in terms of annotation prediction. However, MPSS built using Dali distances receive a very large improvement. We postulate that this is most likely due to the sparseness of the Dali alignment data, which arises due to Dali's stringent requirements for a significant alignment. When stress is minimized, the missing alignments (assigned the maximum distance value) cause unrelated structures to move apart, while the remaining highly significant alignments pull groups of related structures together. Conversely, stress minimization actually decreases the accuracy of classification using CE-based MPSS. We hypothesize that the rigid CE distances contain a high degree of noise. Classical MDS, by discarding eigenvectors with low signal-to-noise, reduces the impact of this noise on the MPSS coordinates, but stress minimization then results in over-fitting as the coordinates are deformed to match the (noisy) pairwise alignment data. Nevertheless, the magnitude of this effect is small.

### Structural diversity of evolutionarily related proteins

The assignment of a set of proteins to the same SCOP family, SCOP superfamily or CATH homologous superfamily is based on the inference of a common evolutionary origin. Such inferences are drawn on the basis of automatic and/or manual inspection of primary, secondary and tertiary structure. Disregarding any effects of subjectivity on classification, it is to be expected that different sets of evolutionarily related proteins will be characterized by varying levels of structural diversity, due primarily to biochemical constraints on the ability of different structures to provide the same or similar functions. The structural diversity of any group of proteins may be examined quantitatively using structural distances from algorithmic structure alignments. Correct assessment of the relative structural diversity of a group requires examining both the distribution of distances within the group, and that of distances between group members and all other protein structures. We visualize these distributions by constructing within-group and global distance histograms for the three annotations (SCOP family, SCOP superfamily and CATH homologous superfamily) which convey an evolutionary relationship. The variation of structural diversity within each type of group is immediately apparent. Groups with high degrees of structural self-similarity have within-group distributions which peak relatively near the zero distance and then fall off as distance increases. In contrast, structurally diverse groups have within-group distance distributions which peak far from zero, and are similar to the distribution of distances between group members and all other structures. Groups with intermediate levels of structural diversity have within-group histograms between these two extremes.

**Figure 4:**
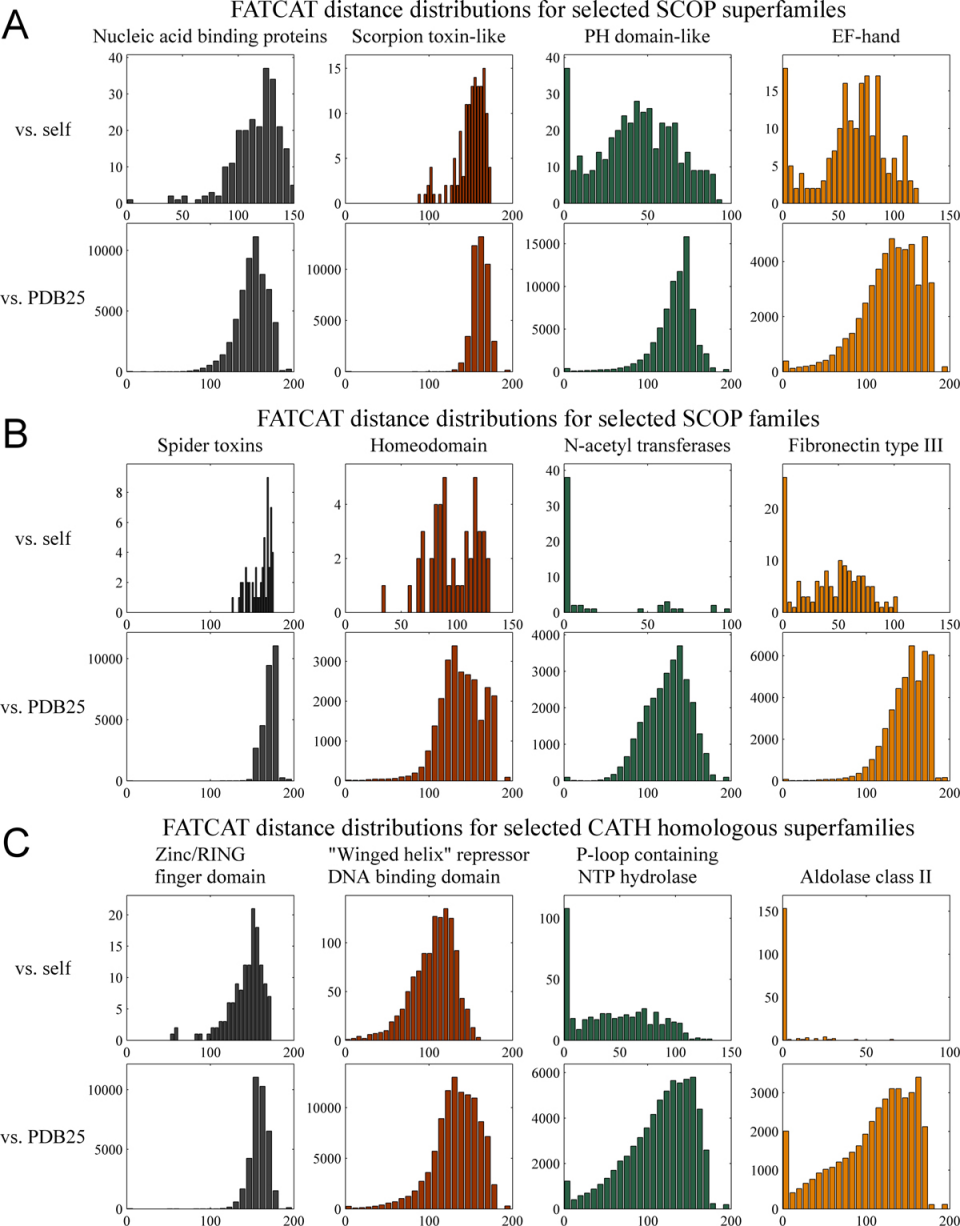
**Distance distributions of diverse and self-similar annotation groups**. Each part displays histograms of FATCAT alignment distance between members of a group and all other structures (bottom, "vs. PDB25") and between group members only (top, "vs. self"). The left two groups for each classification level are considered structurally diverse, while the right two are considered structurally self-similar. SCOP superfamily, SCOP family and CATH superfamily ("H") (Parts (A)-(C) respectively) were selected because they are based on shared function or evolutionary origin rather than structure alone.

Figure 4 presents histograms showing the distribution of raw FATCAT distances for diverse and self-similar groups within each annotation type. FATCAT distances are used because of the extreme sparseness of the data from Dali and because of their better predictive capability compared to CE distance (*vide infra*). The figure demonstrates that groups of structures believed to have common evolutionary origins are characterized by varying structural diversity (as could be expected) and that groups can be classified as "diverse" or "self-similar" by inspecting their global and within-group distance distributions. In particular, we propose a simple decision function for discriminating between such groups for the purposes of further analysis. We classify a group as homogeneous or self-similar if the most populated histogram bin is at zero, and as diverse if the histogram mode is far (greater than 50 FATCAT distance units) from zero. For the purposes of the present work, groups of intermediate diversity need not be considered.

### Impact of structural diversity on annotation inference

**Figure 5:**
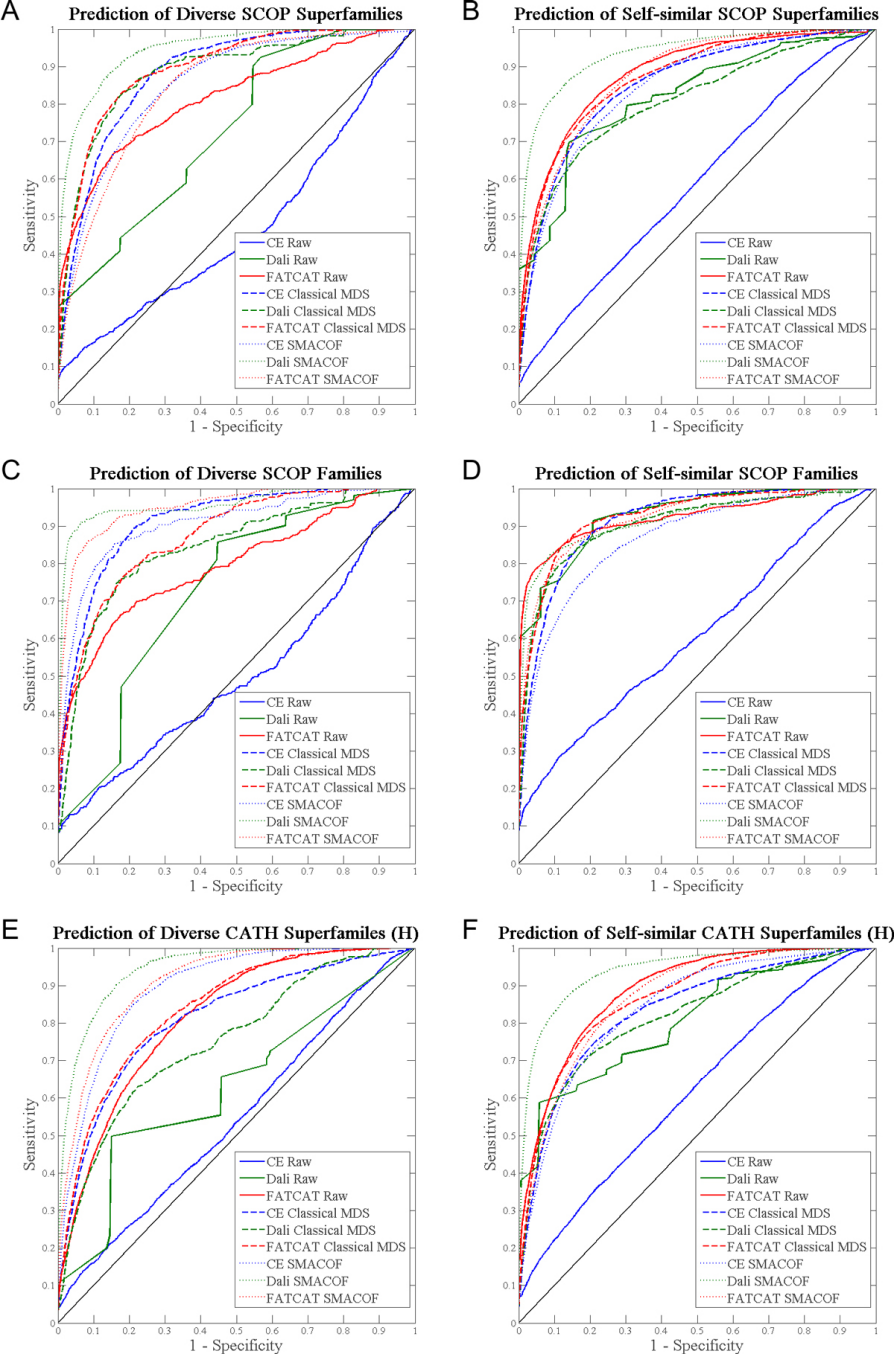
**Differential classification performance for diverse and self-similar annotation groups**. The left column shows ROC for prediction of annotation within diverse groups of the three classification types, using pairwise distances from all three alignment methods and both MDS methods, and the right column shows ROC curves for prediction of annotation within homogeneous or self-similar groups. While MPSS proximity is an effective classifier for both diverse and self-similar groups of structures, pairwise distances do not perform well for diverse groups, regardless of alignment method. MPSS produced using SMACOF are especially successful for diverse groups, but may very slightly over-fit the positions of structures within homogeneous groups.

Does the structural diversity of a group of proteins affect the ability of pairwise alignments or MPSS proximity to predict group membership? We address this question by computing independent ROC curves for the most populated examples of structurally homogeneous and structurally diverse groups (as identified in Table 1). These data are shown in Figure 5, in which ROC plots for diverse and self-similar groups belonging to SCOP superfamiles, SCOP families and CATH superfamilies are presented side-by-side. Although pairwise alignments and MPSS proximity can be seen to perform similarly for self-similar groups (as they do for all groups on average), pairwise alignments lose much of their predictive ability for annotation of common evolutionary origin when applied to structurally diverse groups. In contrast, MPSS representations - especially those computed using SMACOF - achieve nearly the same, and in some cases higher, levels of performance for diverse groups than for homogeneous ones.

## Conclusions

We found that the qualitative shape of a MPSS is highly dependent on the alignment method used for distance estimation. Quantitative performance in terms of annotation prediction is also dependent on both alignment and MDS method. On average among all protein groups, flexible alignment using FATCAT obtains high performance for both scaling approaches, while the Dali/SMACOF pairing produces the highest overall accuracy. For highly diverse groups, the best performance is obtaining using SMACOF, again particularly in combination with Dali. Furthermore, while both MPSS and pairwise distances have predictive capability for annotations of shared function or origin, only MPSS retain this predictive power for those which are highly structurally diverse.

We are currently developing a publicly available web server called PSPACE where users can create MPSS by selecting alignment methods, varying different parameters and subsequently use the resultant maps to study existing as well as novel structures.

## List of abbreviations

CATH: Class, Architecture, Topology, Homology; FPR: False Positive Rate; MDS: Multidimensional Scaling; MPSS: Map(s) of Protein Structure Space; PDB: Protein Data Bank; PSS: Protein Structure Space; ROC: Receiver Operating Characteristic; SCOP: Structural Classification of Proteins; SMACOF: Scaling by Majorizing a Complex Function; TPR: True Positive Rate.

## Competing interests

The authors declare that they have no competing interests.

## Authors' contributions

R. Singh conceived the research. The algorithmic and experimental design was done by D. Asarnow and R. Singh. D. Asarnow generated the data, implemented methods and conducted experiments. D. Asarnow and R. Singh jointly analyzed the experimental findings and composed the manuscript.

## Declarations

Publication of this work was supported by the National Science Foundation grant IIS0644418 (CAREER).

This article has been published as part of *BMC Proceedings *Volume 7 Supplement 7, 2013: Proceedings of the Great Lakes Bioinformatics Conference 2013. The full contents of the supplement are available online at http://www.biomedcentral.com/bmcproc/supplements/7/S7.
